# Invasive Lobular Cancer Arising in a Surgical Scar From Lumpectomy for a Previous Invasive Ductal Cancer of the Breast

**DOI:** 10.7759/cureus.29054

**Published:** 2022-09-11

**Authors:** Richard Adam, Mira Herman, Laura Hodges, Tim Q Duong, Susan Fineberg, Shima Roknsharifi

**Affiliations:** 1 Radiology, Stony Brook University, Stony Brook, USA; 2 Radiology, Montefiore Medical Center/Albert Einstein College of Medicine, Bronx, USA; 3 Pathology, Montefiore Medical Center/Albert Einstein College of Medicine, Bronx, USA

**Keywords:** histologically new, invasive ductal carcinoma, invasive lobular carcinoma, scar, breast cancer

## Abstract

We describe a case of pathology-proven invasive lobular breast cancer (ILC) arising in a scar over 15 years after lumpectomy for previous invasive ductal carcinoma (IDC). The tumor was detected on screening mammography as a new focal asymmetry at the scar site and confirmed at diagnostic mammography. Ultrasound demonstrated an irregular, shadowing, hypoechoic mass at the scar site. Ultrasound-guided biopsy revealed poorly differentiated invasive lobular carcinoma. MRI and CT showed an irregular mass with pectoralis muscle invasion. Multimodality imaging findings are described. This is the first case to our knowledge reporting multimodality imaging findings of a breast cancer developing at the site of a surgical scar that is histologically different from the originally resected cancer.

## Introduction

Breast cancer arising in a scar is an unusual occurrence with few previous reports. The earliest of these reports dates back to 1968, and in that report, an association between benign breast biopsies and subsequent breast cancers was demonstrated [1]. Eight years later, a series of 12 cases of breast cancer arising in surgical scars from benign etiologies was published [2]. A more recent case published in 2007 describes a cancer arising in a surgical scar from benign excision with associated ultrasound findings [3]. Cancers arising in scars in other anatomical locations have been widely described in the literature, with well-established pathophysiological mechanisms for their occurrence [4-9]. We describe a case of breast cancer arising in a scar from a previously distinct breast cancer of different histology. We present the first report of multimodality imaging findings of breast cancer arising in a scar and provide a review of the literature.

## Case presentation

**Figure 1:**
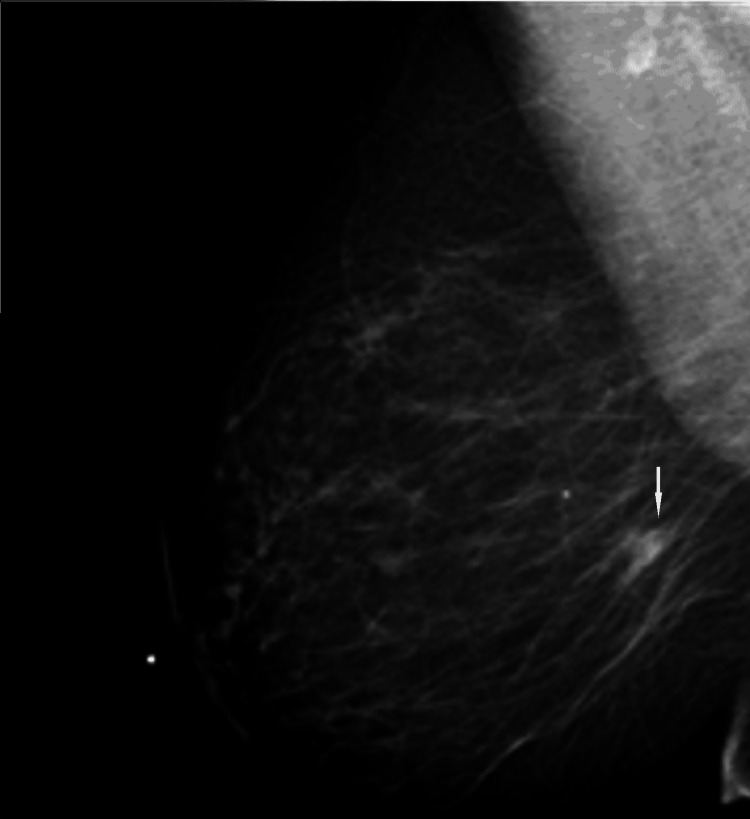
MLO view from screening mammogram 16 years prior to presentation. There is a focal asymmetry in the central inner right breast at posterior depth. This focal asymmetry was biopsied, and pathology revealed stage IA estrogen receptor-positive/progesterone receptor-positive/human epidermal growth factor receptor 2-negative (ER+/PR+/HER2-) invasive ductal carcinoma and ductal carcinoma in situ (DCIS). MLO: mediolateral oblique

**Figure 2:**
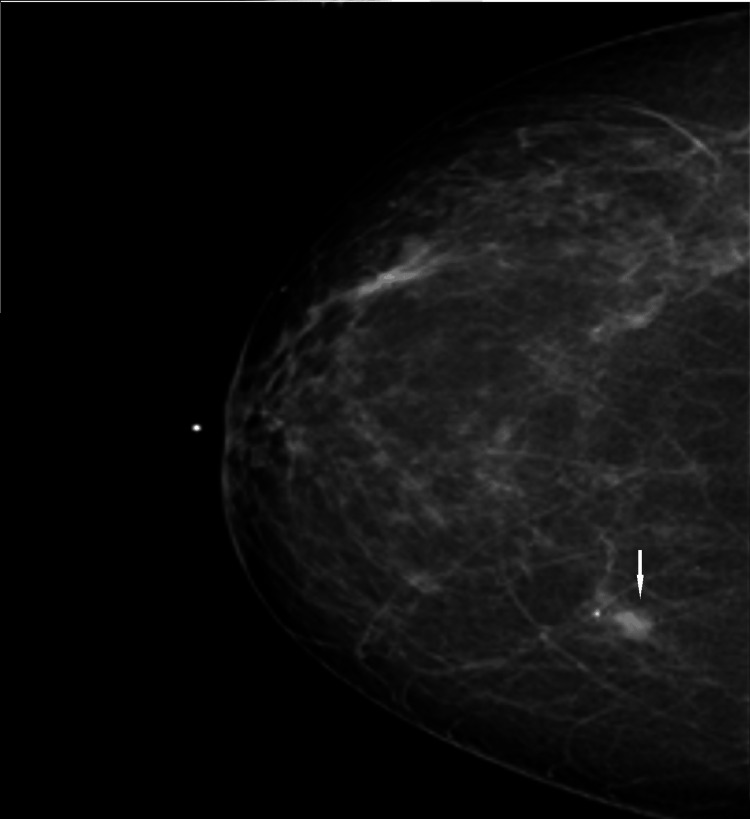
CC view from screening mammogram 16 years prior to presentation. There is a focal asymmetry in the central inner right breast at posterior depth. This focal asymmetry was biopsied, and pathology revealed stage IA estrogen receptor-positive/progesterone receptor-positive/human epidermal growth factor receptor 2-negative (ER+/PR+/HER2-) invasive ductal carcinoma and ductal carcinoma in situ (DCIS). CC: craniocaudal

**Figure 3:**
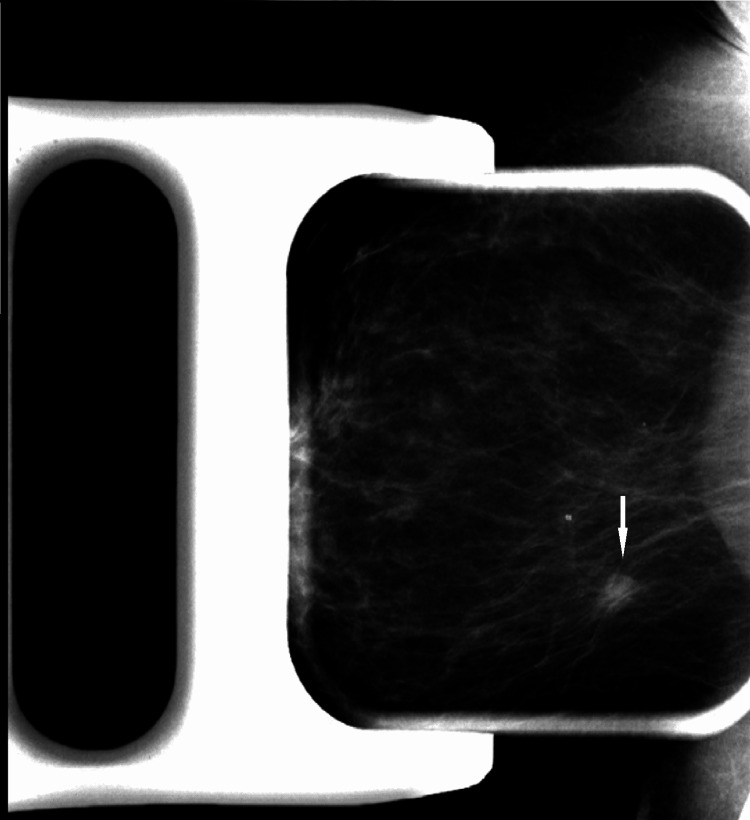
Spot compression diagnostic mammogram 16 years prior to presentation. The focal asymmetry in the central inner right breast at posterior depth persists on spot compression view. This focal asymmetry was biopsied, and pathology revealed stage IA estrogen receptor-positive/progesterone receptor-positive/human epidermal growth factor receptor 2-negative (ER+/PR+/HER2-) invasive ductal carcinoma and ductal carcinoma in situ (DCIS).

A woman in her seventh decade presented for screening mammography. She had a history of lumpectomy, chemotherapy, and radiation therapy for stage IA estrogen receptor-positive/progesterone receptor-positive/human epidermal growth factor receptor 2-negative (ER+/PR+/HER2-) invasive ductal carcinoma (IDC) and ductal carcinoma in situ (DCIS) originally diagnosed 16 years prior to presentation. Screening and diagnostic mammograms at that time demonstrated a focal asymmetry in the central inner right breast at posterior depth (Figures 1-3), which was further biopsied.

**Figure 4:**
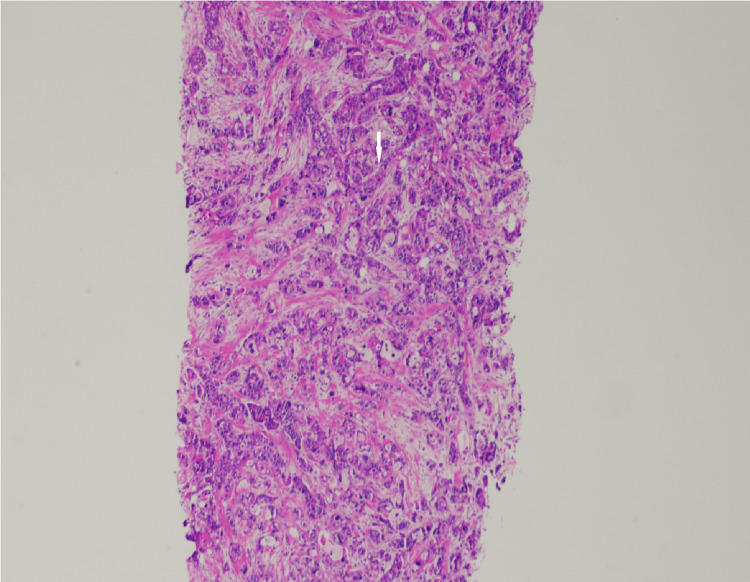
Low-power (×40) H&E stain 16 years prior to presentation. Pathology demonstrates poorly differentiated invasive ductal carcinoma (IDC) evident by high nuclear grade within cell nests (Nottingham grade: 8/9, tubule score: 3/3, nuclear grade: 3/3, mitotic index: 2/3). This tumor was estrogen receptor-positive/progesterone receptor-positive/human epidermal growth factor receptor 2-negative (ER+/PR+/HER2-).

**Figure 5:**
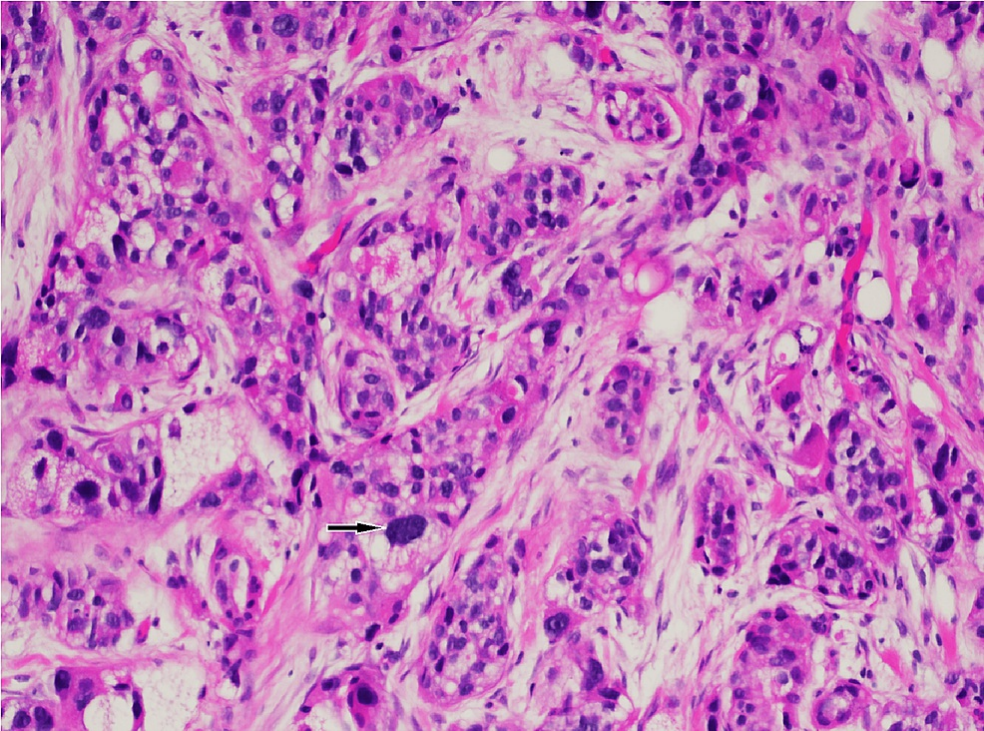
High-power (×200) H&E stain 16 years prior to presentation. Pathology demonstrates poorly differentiated invasive ductal carcinoma (IDC) evident by high nuclear grade within cell nests (Nottingham grade: 8/9, tubule score: 3/3, nuclear grade: 3/3, mitotic index: 2/3). This tumor was estrogen receptor-positive/progesterone receptor-positive/human epidermal growth factor receptor 2-negative (ER+/PR+/HER2-).

Pathology revealed poorly differentiated IDC, which was evident by high nuclear grade within cell nests (Nottingham grade: 8/9, tubule score: 3/3, nuclear grade: 3/3, mitotic index: 2/3). This tumor was estrogen receptor-positive/progesterone receptor-positive/human epidermal growth factor receptor 2-negative (ER+/PR+/HER2-) (Figures 4, 5).

**Figure 6:**
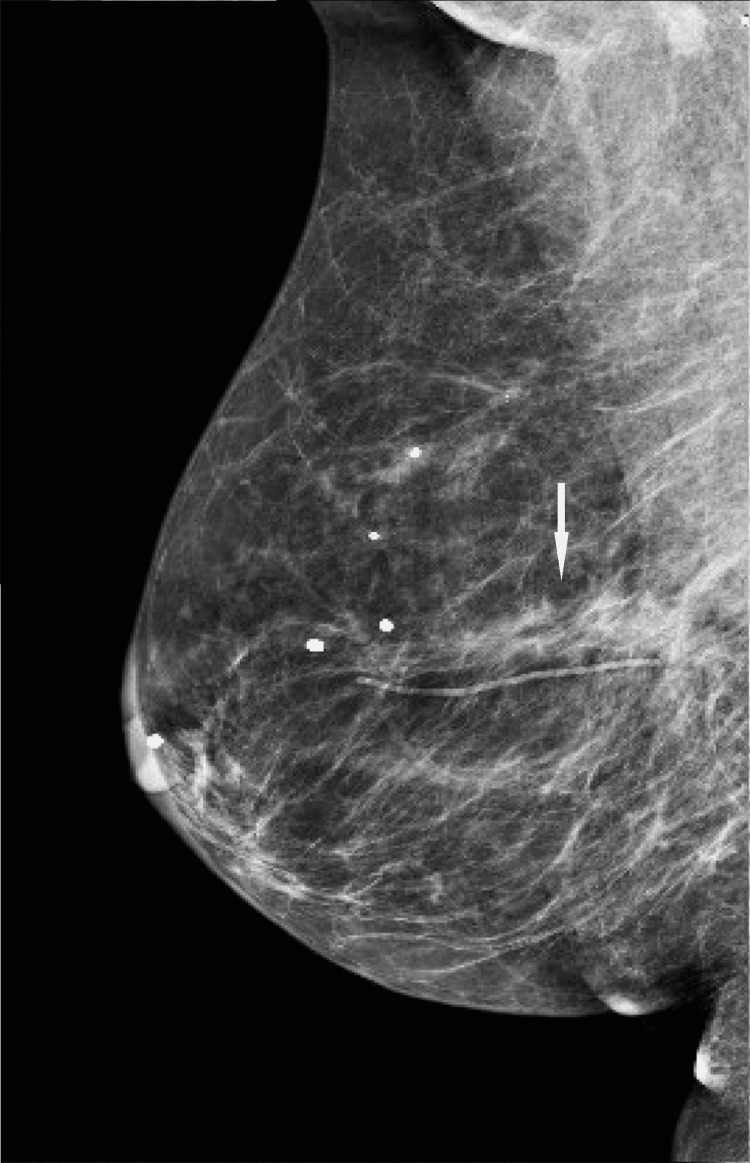
MLO view from screening mammogram 13 years after original diagnosis. There are post-treatment changes in the central inner right breast at posterior depth, which were stable for many years. MLO: mediolateral oblique

**Figure 7:**
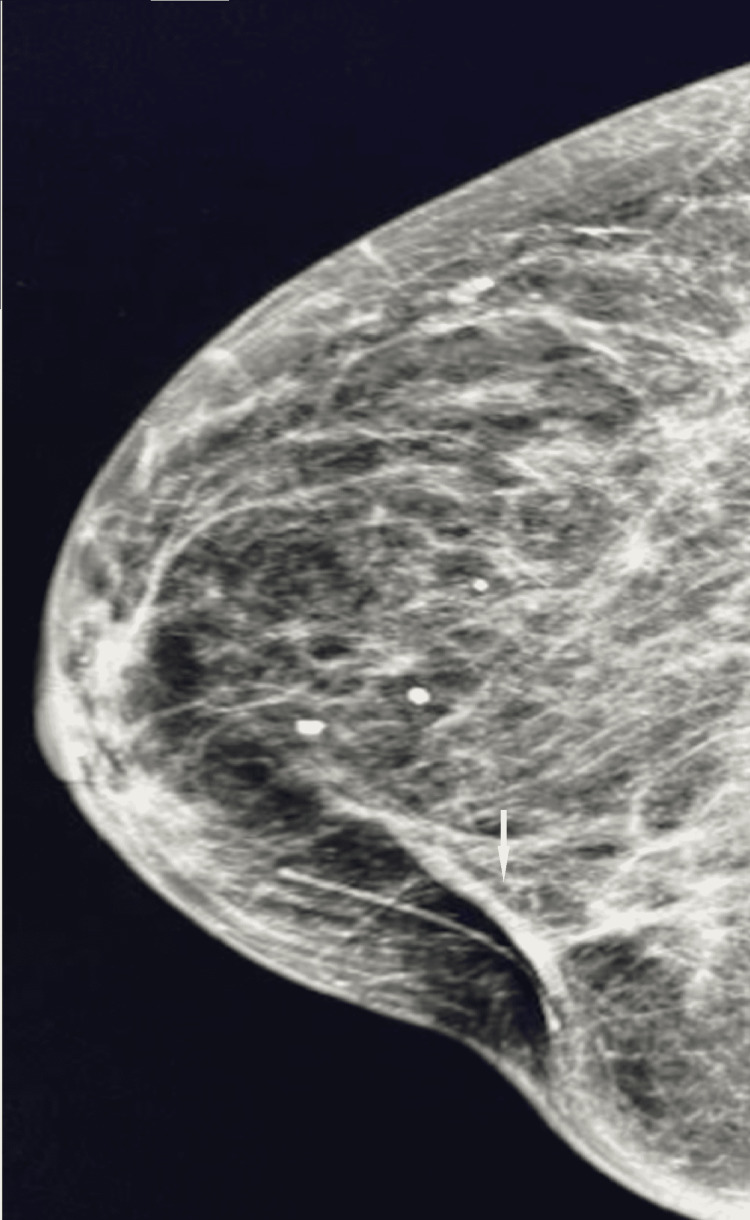
CC view from screening mammogram 13 years after original diagnosis. There are post-treatment changes in the central inner right breast at posterior depth, which were stable for many years. CC: craniocaudal

Following treatment, the post-lumpectomy site was stable mammographically for many years. Screening mammogram from three years prior to presentation (13 years after original diagnosis) is presented as a representative (Figures 6, 7).

**Figure 8:**
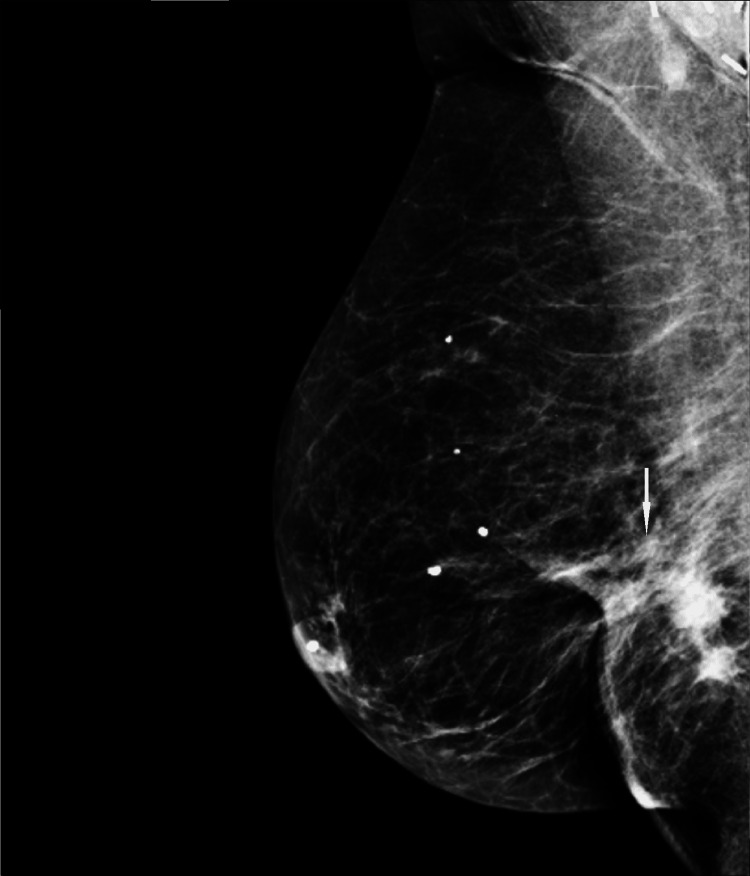
MLO view from screening mammogram at the time of presentation. There is increasing density in the region of the scar in the central inner right breast at posterior depth. MLO: mediolateral oblique

**Figure 9:**
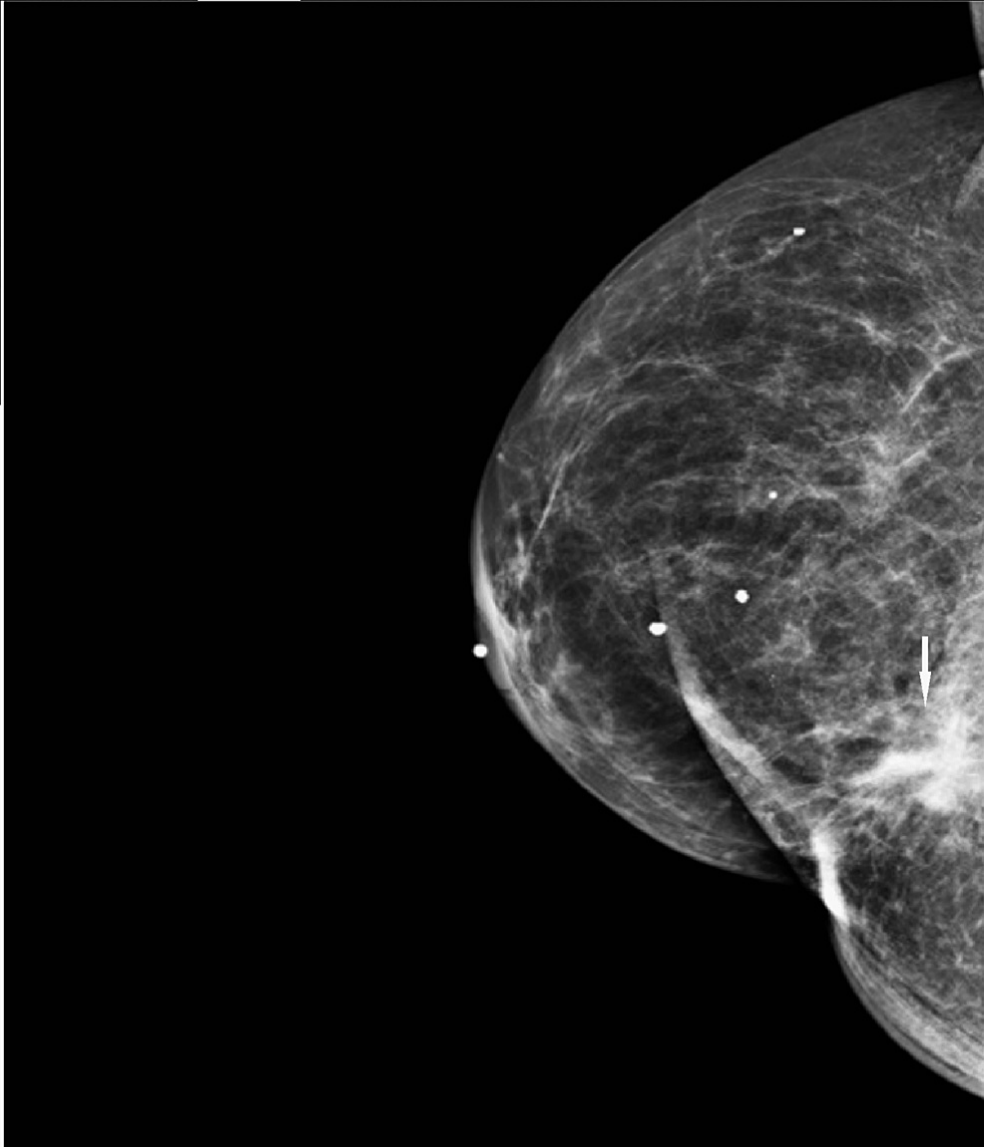
CC view from screening mammogram at the time of presentation. There is increasing density in the region of the scar in the central inner right breast at posterior depth. CC: craniocaudal

During screening mammography at the time of presentation, a new focal asymmetry was identified at the scar site of the original cancer (Figures 8, 9).

**Figure 10:**
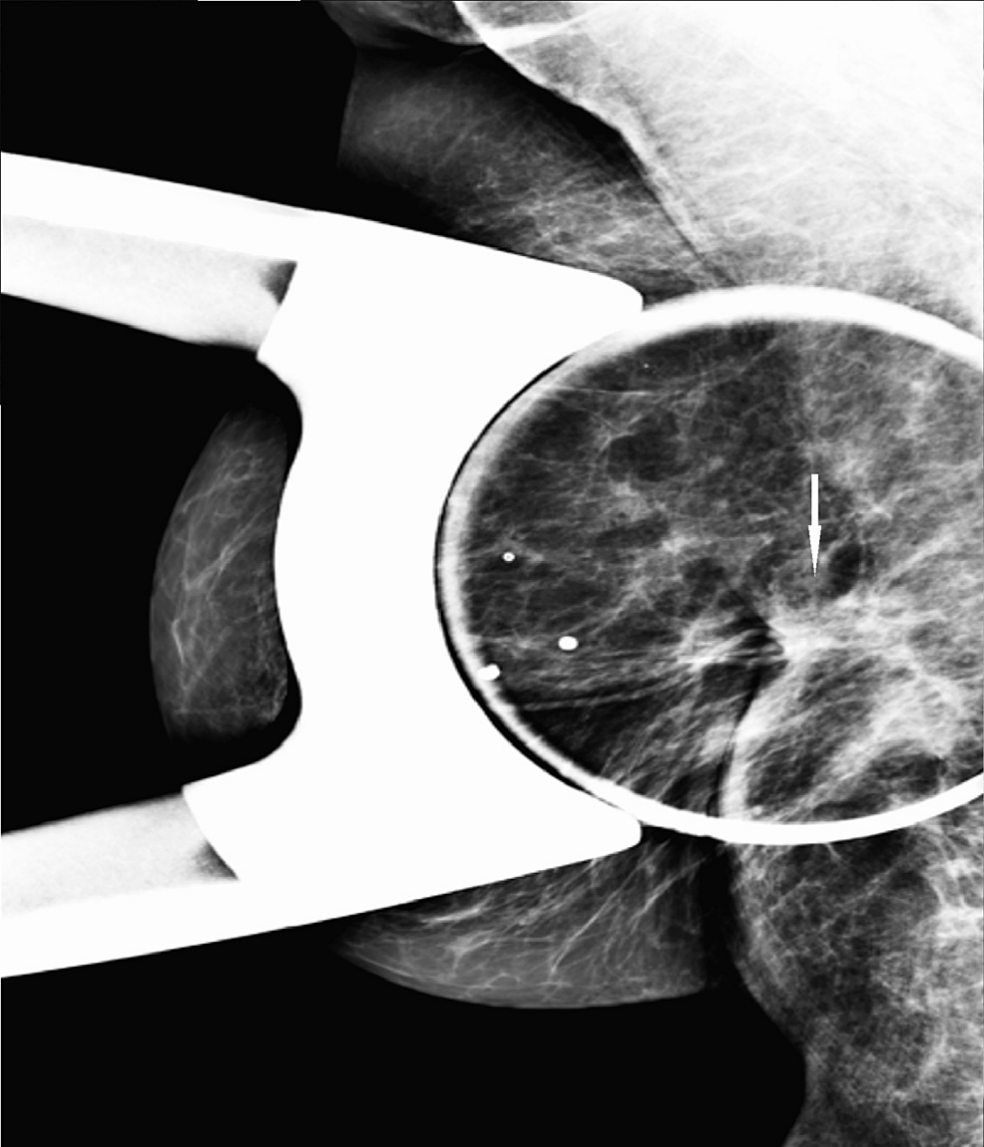
Spot compression diagnostic mammogram (MLO view) at the time of presentation. New increasing density at scar site persists on spot compression view. MLO: mediolateral oblique

Diagnostic evaluation with spot compression diagnostic mammogram and ultrasound was performed. Diagnostic mammogram demonstrated a new increasing density at the scar site (Figure 10).

**Figure 11:**
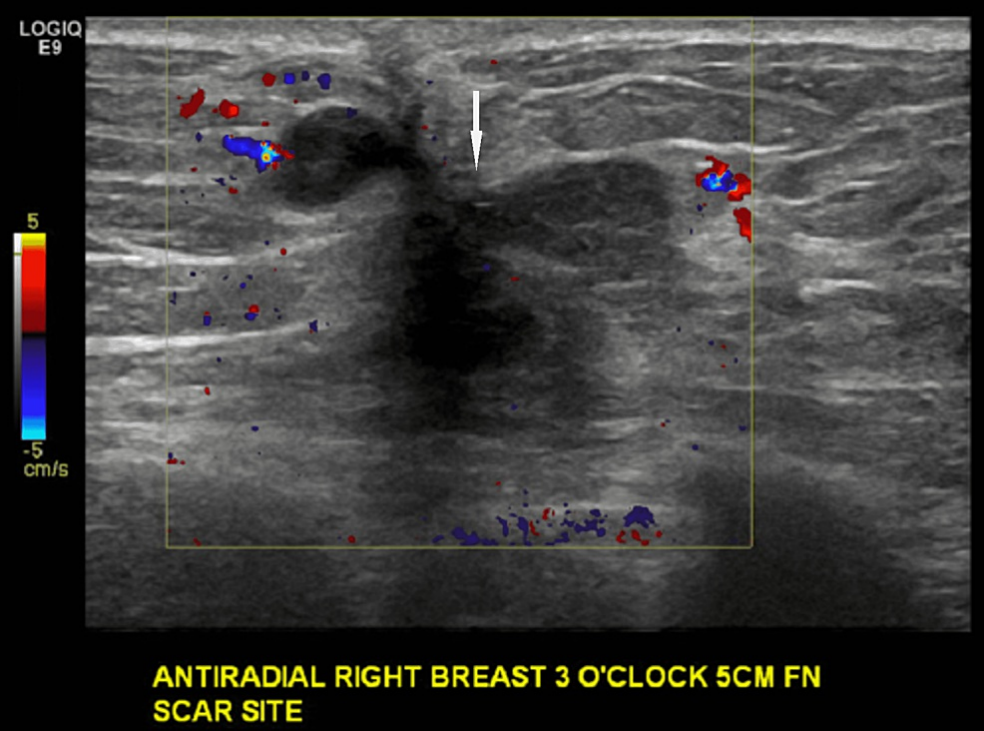
Targeted ultrasound of the right breast at the site of mammographic finding and surgical scar at the time of presentation. Corresponding to the mammographic finding of new increasing density at the site of the scar, there is an irregular, hypoechoic, shadowing mass at three o'clock 5 cm from the nipple.

**Figure 12:**
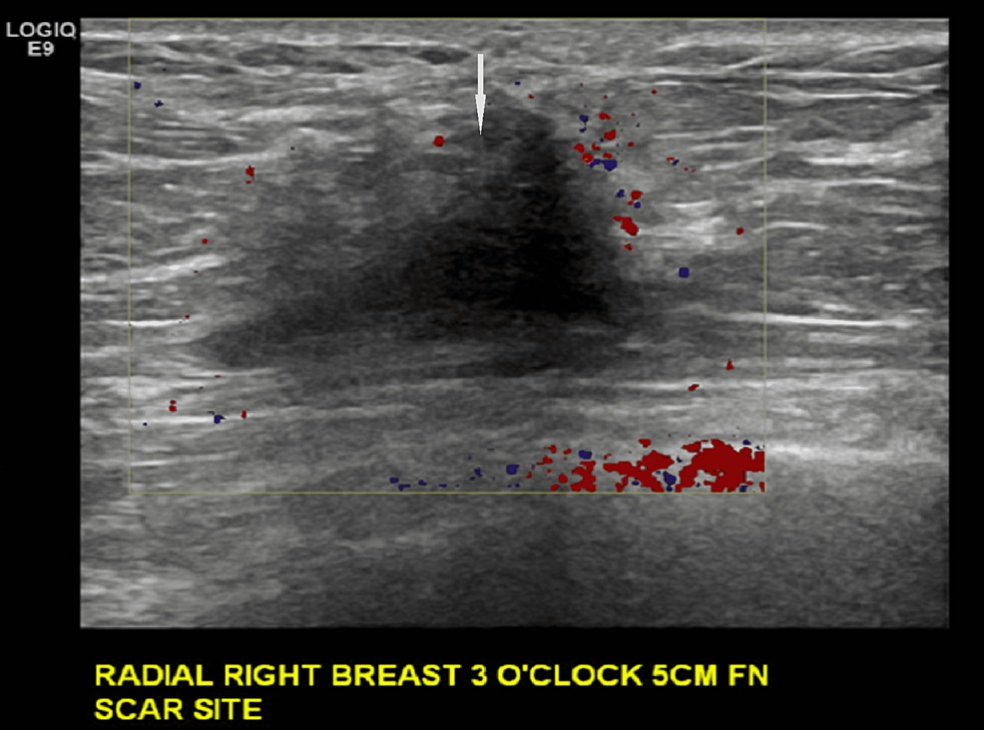
Targeted ultrasound of the right breast at the site of mammographic finding and surgical scar at the time of presentation. Corresponding to the mammographic finding of new increasing density at the site of the scar, there is an irregular, hypoechoic, shadowing mass at three o'clock 5 cm from the nipple.

Targeted ultrasound demonstrated a corresponding hypoechoic, irregular, shadowing mass at the scar site (Figures 11, 12).

**Figure 13:**
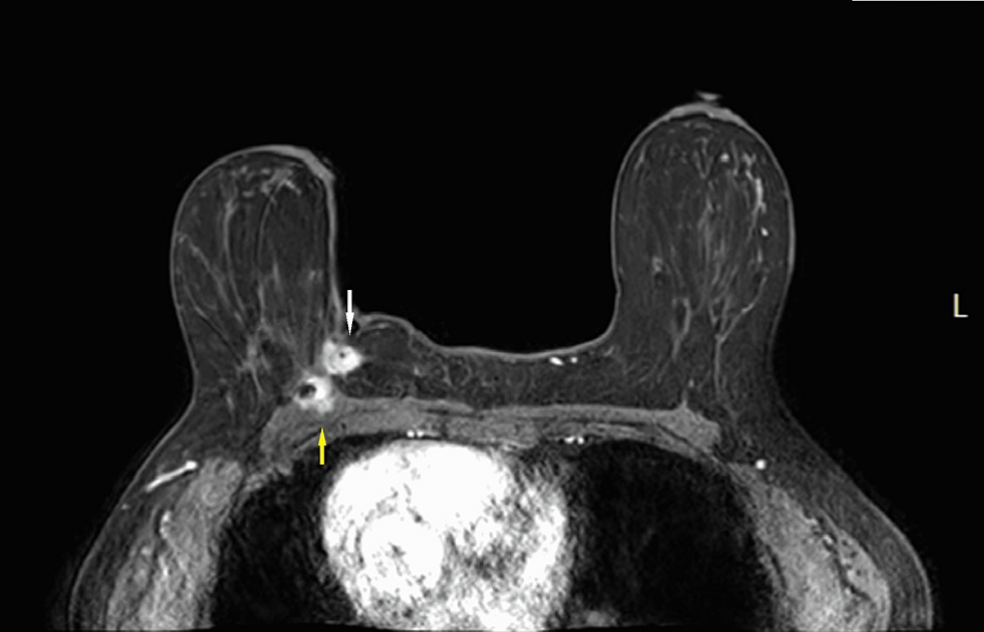
Post-contrast T1 fat-saturated MRI axial image at the time of presentation. Corresponding to the mammographic and ultrasound findings at the site of the surgical scar, there is an enhancing mass in the central inner right breast at posterior depth (white arrow) with pectoralis muscle invasion (yellow arrow).

**Figure 14:**
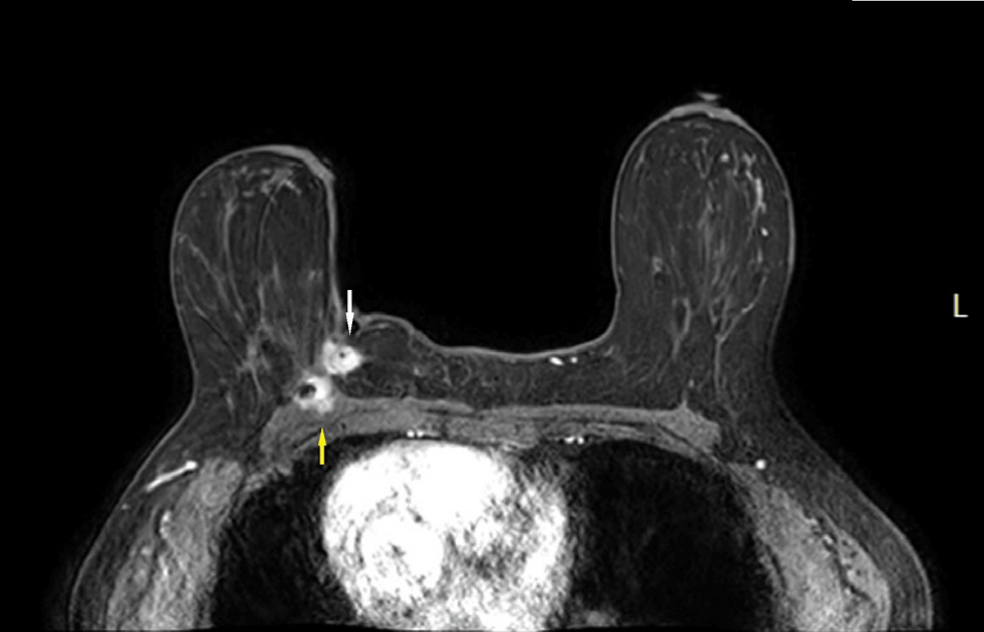
Maximum intensity projection (MIP) MRI image. Corresponding to the mammographic and ultrasound findings at the site of the surgical scar, there is an enhancing mass in the central inner right breast at posterior depth (white arrow) with pectoralis muscle invasion (yellow arrow).

MRI was also performed, which showed an enhancing, irregular mass at the scar site invading the pectoralis muscle (Figures 13, 14).

**Figure 15:**
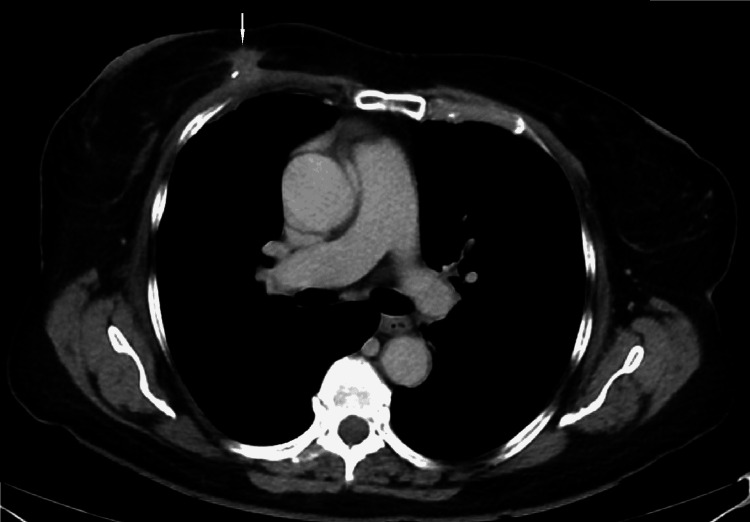
Contrast-enhanced CT at the time of presentation. Contrast-enhanced CT performed for staging demonstrates a mass in the central inner right breast at posterior depth inseparable from the right pectoralis musculature.

This mass was also apparent on a staging CT (Figure 15).

**Figure 16:**
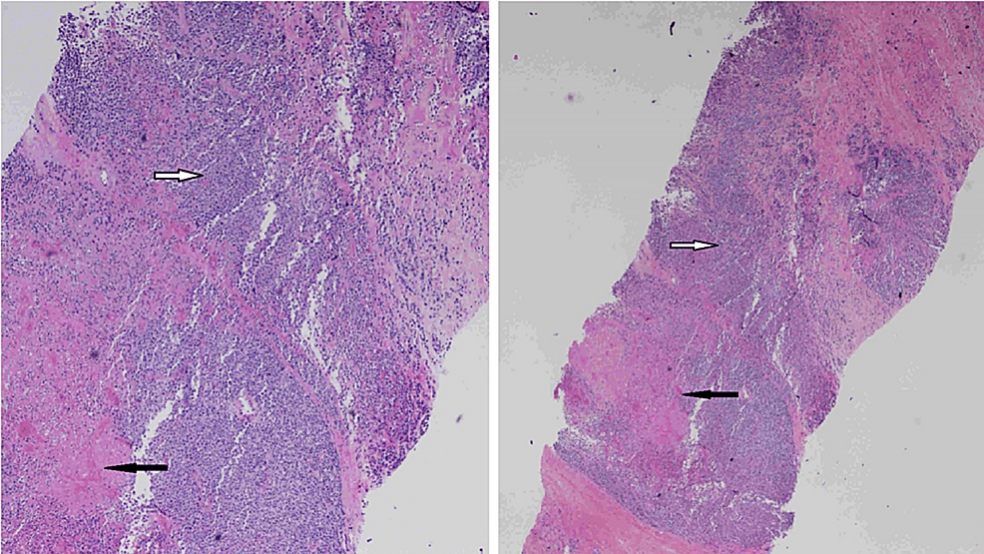
Low-power (×40) H&E stain (right) and high-power (×100) H&E stain (left) at the time of presentation. Pathology demonstrates high degree of cellularity (white arrow) representing poorly differentiated invasive lobular carcinoma (ILC) within surrounding areas of bland fibrous tissue (black arrow). This tumor was estrogen receptor-positive/progesterone receptor-positive/human epidermal growth factor receptor 2-negative (ER+/PR+/HER2-).

**Figure 17:**
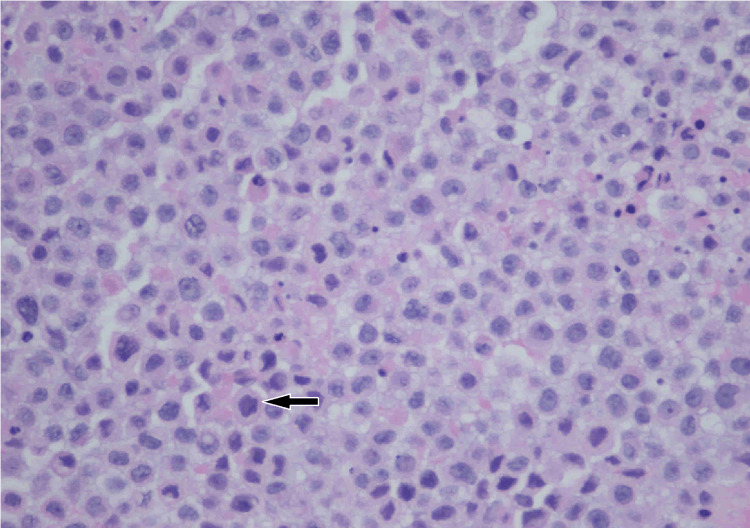
High-power (×400) H&E stain at the time of presentation. There is an abnormal nucleus with high nuclear grade and high degree of cellularity representing poorly differentiated invasive lobular carcinoma (ILC). This tumor was estrogen receptor-positive/progesterone receptor-positive/human epidermal growth factor receptor 2-negative (ER+/PR+/HER2-).

**Figure 18:**
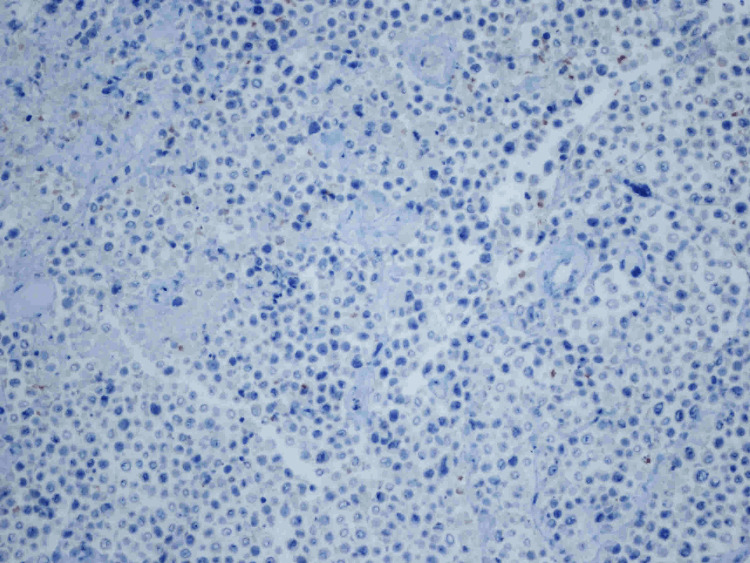
E-cadherin staining (×200) at the time of presentation. E-cadherin is a cell surface receptor protein involved in cell adhesion, which is known to be expressed in ductal carcinoma. However, there is complete loss of expression of this protein in lobular carcinoma. The following image displays negative E-cadherin staining, which confirms that this is not ductal carcinoma but rather lobular carcinoma.

Ultrasound-guided core biopsy at the time of presentation yielded ER+/PR+/HER2- poorly differentiated invasive lobular carcinoma (ILC). The original tumor 16 years prior to presentation was infiltrating ductal carcinoma. However, the tumor arising in the scar at the time of presentation was infiltrating lobular carcinoma (Figures 16, 17). There was negative staining for E-cadherin from the biopsy of the tumor in the scar (Figure 18). E-cadherin is a cell surface receptor protein involved in cell adhesion, which is known to be expressed in ductal carcinoma. However, there is complete loss of expression of this protein in lobular carcinoma as in this case.

 

## Discussion

There is a considerable body of literature describing cancers arising from scars in anatomical locations outside of the breast. Chronic inflammation is an established risk factor for malignant transformation with the oldest documented report dating back to 1907 by Segond and others [4-9]. Several theories have been proposed for the pathogenesis of carcinogenesis in scar tissue. Scarring promotes fibrosis, a relatively avascular environment, and a prolonged healing process subject to minor trauma and repeated repair. This may set the stage for dysplastic changes [10]. Once tumor cells arise, their growth may be further fueled by the lack of immunologic surveillance, as scar tissue obliterates lymphatic channels [11-13].

The first study examining an association between breast biopsy/surgery and subsequently developing breast cancer was published in 1968. In this paper, Potter et al. documented an increased incidence of breast cancer many years following a benign breast biopsy [1]. These diagnoses appeared to be related to years at risk and not a specific pathologic process associated with the benign pathology result. In 1976, Freund et al. reported 12 women who developed breast cancer at the site of old surgical scars: six occurring from biopsies, three from abscess drainages, and three from thoracotomy scars [2]. The authors proposed several criteria to establish trauma as the inciting factor for malignancy: 1) surgery for an unrelated condition, 2) integrity of the breast preoperatively, 3) complete healing of the surgical wound, 4) tumor site corresponding to scar site, and 5) a reasonable time interval between surgery and occurrence of tumor [2]. In 2007, Kim et al. described breast cancer arising in a scar from the excision of a benign mass (intraductal papilloma). The report catalogues the evolution of a malignancy on sequential ultrasound imaging, which initially appeared as improving fat necrosis after the excisional biopsy. However, after two years, more angular margins and penetrating vascularity developed [3].

There are relatively few case reports describing this phenomenon in the breast. Our case satisfies Freund et al.'s criteria and serves to contribute further to this body of literature. The second cancer that our patient developed at the same site occurred over 15 years after the initial surgery and was a pathologically distinct diagnosis from the original cancer. The change in appearance on mammography and corresponding ultrasound findings was suspicious, particularly given the previous chronic imaging stability. Post-contrast MRI and CT also documented the presence of this malignancy at the scar site, with the invasion of the pectoralis muscle.

The differential diagnosis for a second breast malignancy at the site of previous breast cancer includes recurrence and radiation-induced malignancy. Recurrence would be the most likely diagnosis, and in this scenario, the new malignancy would be pathologically similar to the original tumor. This clearly was not the case with our patient. Radiation-induced malignancy can occur when radiation therapy was given for the first tumor, as is typical in patients undergoing breast conservation surgery. These tumors typically present 15+ years following radiation therapy [14]. Sarcomas, particularly angiosarcoma, are mainly associated with this risk factor [15]. While the patient presented in this report was exposed to radiation therapy and presented at a sufficient time interval post treatment for a radiation-induced sarcoma, the histology of the malignancy (invasive lobular carcinoma) is not consistent with this etiology. The relative risk for postradiation second primary breast cancer is increased at 5-10 years [14]. This does not correspond to the time frame at which our patient developed a second breast cancer, and therefore, we do not favor this etiology in our patient. A third differential diagnosis is a scar-associated malignancy, which we favor as the etiology in this case.

## Conclusions

This is the first reported case to our knowledge of a histologically different breast cancer, which was ILC developing at the site of a surgical scar from a previous IDC with multimodality imaging findings. Although recurrence in the surgical bed is not uncommon, a de novo cancer of a different histological type than the original cancer in the surgical bed is unusual. Chronic inflammation is a known risk factor for malignant transformation. Specifically, the pathophysiology of carcinogenesis in scar tissue likely explains the occurrence of a histologically different tumor occurring in the surgical bed. A new cancer in the original tumor bed secondary to a scar should be considered in the differential diagnosis when imaging findings reveal a mass occurring in a previously stable area of scarring for several years.
